# Resistance Exercise Program in Cognitively Normal Older Adults: CERT-Based Exercise Protocol of the AGUEDA Randomized Controlled Trial

**DOI:** 10.1007/s12603-023-1982-1

**Published:** 2023-09-30

**Authors:** Beatriz Fernandez-Gamez, P. Solis-Urra, M. Olvera-Rojas, C. Molina-Hidalgo, J. Fernández-Ortega, C.P. Lara, A. Coca-Pulido, D. Bellón, A. Sclafani, J. Mora-Gonzalez, A. Toval, I. Martín-Fuentes, E.A. Bakker, R.M. Lozano, S. Navarrete, D. Jiménez-Pavón, T. Liu-Ambrose, K.I. Erickson, F.B. Ortega, Irene Esteban-Cornejo

**Affiliations:** 1 Department of Physical Education and Sports, Faculty of Sport Sciences, Sport and Health University Research Institute (iMUDS), University of Granada, Carretera de Alfacar, 21, 18071, Granada, Spain; 2 Servicio de Medicina Nuclear, Hospital Universitario Virgen de las Nieves, 18014, Granada, Spain; 3 Faculty of Education and Social Sciences, Universidad Andres Bello, 2531015, Viña del Mar, Chile; 4 Department of Psychology, University of Pittsburgh, Pittsburgh, PA, USA; 5 Neuroscience Institute, AdventHealth Research Institute, Orlando, Florida, USA; 6 Junta de Andalucía, Andalusian Centre of Sport Medicine (CAMD), Granada, Spain; 7 MOVE-IT Research Group, Department of Physical Education, Faculty of Education Sciences and Biomedical Research Innovation Institute of Cádiz, University of Cadiz, Andalusia, Spain; 8 CIBER of Frailty and Healthy Aging (CIBERFES), Madrid, Spain; 9 Department of Physical Therapy, Faculty of Medicine, University of British Columbia, Vancouver, BC, Canada; 10 Centre for Aging SMART at Vancouver Coastal Health, Vancouver Coastal Health Research Institute, Vancouver, BC, Canada; 11 Djavad Mowafaghian Centre for Brain Health, Vancouver Coastal Health Research Institute, Vancouver, BC, Canada; 12 Centro de Investigación Biomédica en Red Fisiopatología de la Obesidad y Nutrición (CIBERobn), Instituto de Salud Carlos III, 28029, Madrid, Spain; 13 Faculty of Sport and Health Sciences, University of Jyväskylä, Jyväskylä, Finland; 14 Instituto de Investigación Biosanitaria ibs. GRANADA, Granada, Spain

**Keywords:** Aging, brain health, cognition, physical activity, strength training

## Abstract

**Objectives:**

To provide a comprehensive CERT (Consensus on Exercise Reporting Template)-based description of the resistance exercise program implemented in the AGUEDA (Active Gains in brain Using Exercise During Aging) study, a randomized controlled trial investigating the effects of a 24-week supervised resistance exercise program on executive function and related brain structure and function in cognitively normal older adults.

**Design and Participants:**

90 cognitively normal older adults aged 65 to 80 were randomized (1:1) to a: 1) resistance exercise group; or a 2) wait-list control group. Participants in the exercise group (n = 46) performed 180 min/week of resistance exercise (3 supervised sessions per week, 60 min/session) for 24 weeks.

**Intervention:**

The exercise program consisted of a combination of upper and lower limb exercises using elastic bands and the participant's own body weight as the main resistance. The load and intensity were based on the resistance of the elastic bands (7 resistances), number of repetitions (individualized), motor complexity of exercises (3 levels), sets and rest (3 sets/60 sec rest), execution time (40–60 sec) and velocity (as fast as possible).

**Settings:**

The maximum prescribed-target intensity was 70–80% of the participants' maximum rate of perceived exertion (7–8 RPE). Heart rate, sleep quality and feeling scale were recorded during all exercise sessions. Those in the wait-list control group (n = 44) were asked to maintain their usual lifestyle. The feasibility of AGUEDA project was evaluated by retention, adherence, adverse events and cost estimation on the exercise program.

**Results and Conclusions:**

This study details the exercise program of the AGUEDA trial, including well-described multi-language manuals and videos, which can be used by public health professionals, or general public who wish to implement a feasible and low-cost resistance exercise program. The AGUEDA exercise program seems to be feasible by the high retention (95.6%) and attendance rate (85.7%), very low serious adverse event (1%) and low economic cost (144.23 € /participant/24 weeks). We predict that a 24-week resistance exercise program will have positive effects on brain health in cognitively normal older adults.

## Abbreviation

AGUEDA: Active Gains in brain Using Exercise During Aging
CERT: Consensus on Exercise Reporting Template
HR: Heart rate
ICFSR: International Conference of Frailty and Sarcopenia Research
ACSM: American College of Sports Medicine
IMUDS: University Institute of Sports and Health
RPE: Rate of perceived exertion
STICS-m: Spanish version of the modified Telephone Interview of Cognitive Status
MMSE: Mini-Mental State Examination
MoCA: Montreal Cognitive Assessment
GDS: Geriatric Depression Scale
Rep: Repetition
Sec: Seconds
Cm: Centimeters
In: Inches

## Introduction

**E**xercise is medicine, and as such it has documented beneficial effects on the brain (1). Physical exercise is a promising strategy to prevent cognitive decline (2, 3, 4, 5, 6, 7, 8) and is a treatment for improving global cognition and brain-related health outcomes (4, 9, 10). Various types and doses of exercise likely have their own specific neurophysiological responses (11) and associated cognitive benefits (5). Indeed, certain doses (e.g., 10 METs-h/week) may be required for detecting exercise-induced cognitive changes (12). However, there are still important gaps in knowledge that limit well-designed and targeted exercise programs for health benefits. Some of these identified gaps are (i) lack of information about detailed type and dose of exercise (i.e., what), (ii) potential moderators (i.e., for whom) and (iii) the underlying mechanisms (i.e., how) (13, 14).

Emerging evidence supports the potential role of resistance exercise on brain health in older adults (4, 15, 16, 17, 18, 19, 20, 21, 22). Previous studies showed that resistance exercise may be an effective alternative to other types of exercise such as aerobic (23), high intensity interval training (24) or mind-body exercises (i.e., Tai-chi (25), yoga (26)) for improving cognitive functioning and brain health (9). However, the effects of resistance exercise on brain health remain poorly understood, and this is partly due to the significant heterogeneity in the exercise characteristics employed (9, 19, 27, 28). Previous systematic reviews and meta-analyses in older adults have described the heterogeneity, and inconsistency among intervention's characteristics that greatly limits interpretability and consensus (19, 21, 28). For example, the duration of resistance exercise interventions ranges from 8 weeks up to 1 year (22), the weekly exercise volume ranges from 1 to 3 exercise sessions per week (29) with 30 to 100 min per session (30). In addition, some studies reported 2 to 4 sets (31, 32) of a range from 6 to 20 repetitions (33), including body weight (30), elastic bands (18, 31, 34), dumbbells and barbell exercises (19, 31) or exercise machines (35). Other interventions have taken into account different velocities of execution (i.e., high-low speed) (34). Notably, previous studies provide insufficient intervention characteristics and inconsistent information for replication of resistance exercise programs (14). If exercise is to be prescribed as medicine for the prevention or treatment of neurocognitive problems then we need to precisely describe the intervention needed to modify cognitive and brain health in late adulthood (5, 13, 36, 37).

High-quality and detailed reporting of exercise interventions is needed (14) to improve quality appraisal, enable evidence synthesis and replication, and improve translation of resistance exercise programs with the aim of improving brain health. To address this challenge, the Consensus on Exercise Reporting Template (CERT) provides a standardized format to report exercise intervention programs (38). The CERT guidelines include 16 items as the minimal amount of information necessary to report exercise interventions, and to allow development, guidance, evaluation, interpretation and assistance with an effective exercise program for everyday clinical practice (38).

Collectively, well-designed CERT-based randomized controlled trials (RCTs) should report detailed information about FIIT exercise principles (Frequency, Intensity, Time and Type) to establish accessible (which favors its applicability to any population and context), robust, and replicable evidence-based exercise recommendations for brain health. Thus, the aim of this study is to provide the rationale and comprehensive description, based on CERT guidelines, of the 24-week resistance exercise-based program of the AGUEDA trial, in which the primary outcome is to investigate the effects of a 24-week resistance exercise program on executive function in cognitively normal older adults. This may serve researchers and public health practitioners who would like to implement a feasible and low-cost resistance exercise program with expected positive effects on cognitive and brain outcomes in cognitively normal older adults.

## Methods

### Participants and recruitment

A total of 90 cognitively normal older adults (65-80 years old) from Granada (Spain) participated in the AGUEDA trial. Participants were recruited through the local media (television, radio, newspaper), promotional flyers, announcements to local aging and senior citizen agencies, online sites, and social media. The recruitment process started in March 2021 finishing in May 2022.

Inclusion and exclusion criteria were defined as follows: (i) older adults between 65 – 80 years, (ii) physically inactive (i.e., defined as not participating in any resistance exercise program in the last 6 months or accumulating less than 600 METs-Min/ week by the International Physical Activity Questionnaire (IPAQ) (39), (iii) classified as cognitively normal according to the Spanish version of the modified Telephone Interview of Cognitive Status (STICS-m) (≥ 26 points) (40), Mini-Mental State Examination (MMSE) (≥ 25/30) (41) and Montreal Cognitive Assessment (MoCA) (<71 years, ≥ 24/30, 71–75 ≥ 22/30, >75, 21/30) (42); and (iv) without significant depressive symptoms at baseline according to the Geriatric Depression Scale (GDS) (≥ 15) (43). Detailed information about the study design is available elsewhere (44).

### Randomization

Community-dwelling older adults were randomized into a resistance exercise group (n=46) or a wait-list control group (n=44). Participants assigned to the exercise group attended 3 supervised exercise sessions per week during 24 weeks, while the wait-list control group was asked to maintain their usual lifestyle. Participants assigned to the wait-list control group were given an opportunity to attend the 24-week exercise program after completion of the wait-list period. The trial protocol was in accordance with the principles of the Declaration of Helsinki and was approved by the Research Ethics Board of the Andalusian Health Service (CEIM/CEI Provincial de Granada; #2317-N-19 on May 25th, 2020). All participants provided informed consent once all study details were explained. Recruitment, enrollment, and randomization occurred on a rolling basis.

### Exercise program structure

**Figure 1:**
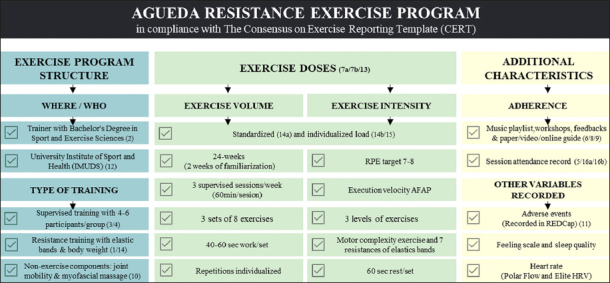
The resistance exercise program implemented in the AGUEDA trial according to the CERT guideline

The 24-week resistance exercise program implemented in the AGUEDA trial followed the CERT guidelines (Table S1) and is summarized in Figure 1. The AGUEDA resistance exercise program was designed based on previous evidence focused on health outcomes (i.e., quality of life or cognitive benefits) in older population (4, 15, 16, 17, 18, 19, 20) and followed the guidelines for resistance training in older adults from the International Conference of Frailty and Sarcopenia Research (ICFSR) (45) and the American College of Sports Medicine (ACSM) (46).

*Numbers in brackets correspond to each CERT item. AFAP:As fast as possible; RPE: Rate of perceived exertion; Sec:Seconds

The resistance exercise program consisted of a combination of upper and lower limb exercises using elastic bands and the participant's own body weight. This enabled easy application, low-cost and feasible translation to different contexts (i.e., homes and clinical practices). The exercise program was performed in groups of 4 – 6 participants and was conducted by professional trainers with a bachelor's degree in Sport and Exercise Sciences. The exercise program was carried out in a fitness room at the University Institute of Sports and Health (IMUDS) in the city of Granada, Spain.

### Exercise equipment

The data collection materials for the training session (i.e., training sheets or adverse event questionnaires) as well as the audiovisual and complementary resources (i.e., exercise program guide) of the exercise program are reposited at GitHub https://github.com/aguedaprojectugr/CERT_AGUEDA. Table S2 shows the list of the files available on the repository.

The elastic bands used were Thera-Band® (47) which are available in 7 different colors corresponding to different resistances (i.e., yellow-soft, red-medium, green-strong, blue-extra strong, black-strong special, silver-athletic and gold-olympic). All the elastic bands have a length of 1.50m and the grip was standardized at 1 meter of length for all participants.

For the execution of exercises with elastic bands, 4 support points were necessary: knee (e.g., woodcutter), waist (e.g., row), head (e.g., face pull) and above the head (e.g., lat pulldown). The participant maintained the maximum elongation distance from the support point without any force on the elastic band. A detailed description of the exercise equipment is shown in Table S3.

### Exercise characteristics and periodization

#### Exercise doses

The total prescribed volume of the AGUEDA trial for each participant assigned to receive exercise was 24 weeks with 3 sessions per week of 60 min duration. Therefore, each participant was prescribed a total of 72 training sessions. The volume is measured by; (i) number of sets (i.e., 3), (ii) number of repetitions (i.e., as many as possible) and (iii) time of set execution (i.e., from 40 to 60 seconds).

Intensity was measured primary by the 10-point Borg Rating of Perceived Exertion scale (RPE) with a target rating of 7–8 (i.e., Very difficult) (48). The Thera-Band RPE scale (47) was also used during the familiarization weeks (i.e., 2 first weeks) to help participants learn the use of the RPE scale with elastics bands. The modifiable variables used to control the intensity were (i) velocity of execution (i.e., as fast as possible), (ii) rest (i.e., 60 seconds of rest each set) and, (iii) levels: basic (level 1), intermediate (level 2) and advanced (level 3). These different levels (8 weeks per level), including 3 training sessions per week/level, progressively increased across the motor complexity of exercises and elastic band resistance (Table 1). Motor complexity involves increasing technical difficulty level of the exercise, which raises the demand for other physical abilities during the resistance exercise stimulating multi-systemic (or multi-component) adaptations (coordination, balance, core stability, power, agility, among others) (49). These multi-systemic adjustments are due to the different characteristics of the resistance exercises: (i) unilateral execution of exercises, which increases the coordination level, and provide changes in the activation pattern of trunk stabilizer muscles (e.g., unilateral push press) (46), (ii) performance of exercises with non-cyclical patterns of movement (e.g., Turkish get-up), which elevates the level of coordination and improves motor control, (iii) multi-segmental exercises that raise the level of stress on the neuromuscular and motor control system (e.g., woodcutter rotation) promoting continuous adaptations (45) or (iv) instability exercise, demanding for balance, joint, and core stability (e.g., walking lunge) (49).

**Table 1:** Training sessions (1, 2 and 3) for each level (1, 2 and 3)

The warm-up (8 minutes) consists of (i) myofascial massage with a tennis ball, (ii) joint mobility, and (iii) movement of the main muscles involved during the training session.				
		**Training session 1 Week 1–8**	**Training session 2 Week 9–16**	**Training session 3 Week 17–24**
**Main-part**	Level 1	Wall push up	Shoulder external rotation *	Bilateral press *
		Lunge	Squat with crossed arms	Lateral lunge
		Standing row*	Standing face pull *	Triceps kickback *
		Standing hip extension on step	Calf-raise	Deadlift *
		Press pallof*	Modified Turkish get-up	Seated shoulder press*
		Lat Pulldown*	Woodcutter*	Hip abduction
		Isometric glute bridge for hamstring	Glute bridge	Modified bird-dog
		Modified dead bug	Kneeling plank	Modified superman
		Incline push up	Shoulder external rotation *	Unilateral press*
		Walking lunge	Squat	Walking lateral lunge
		Standing row*	Standing face pull*	Triceps kickback with inclination *
		Standing hip extension on step*	Single leg standing calf-rise	Deadlift *
	Level 2	Press pallof*	Turkish get-up	Seated shoulder press*
		Lat Pulldown*	Woodcutter rotation *	Side lying hip abduction
		Single leg glute bridge for hamstring	Single leg glute bridge	Bird-dog
		Dead bug	Modified plank	Superman
		Push up	Unilateral shoulder external rotation*	Unilateral press *
		Bulgarian squat	Squat jump	Lateral lunge with elevation
		Standing row*	Standing face pull*	Unilateral triceps kickback with inclination *
		Step-ups	Single leg standing calf-rise on step	Single leg deadlift *
	Level 3	Press pallof*	Turkish get-up with ball	Seated shoulder press*
		Lat Pulldown*	Woodcutter rotation*	Standing hip adduction
		Single leg glute bridge*	Single leg glute bridge for hamstring on step	Advanced bird-dog
		Advanced Dead bug*	Front plank	Swimmer
The cool-down (7 minutes) contains (i) myofascial massage with a tennis ball, (ii) joint mobility, or (iii) stretching which is focussed on the target muscle groups of the training session.				


*Exercises with elastic bands. The rest of exercise are performed with someone's body weight.

Furthermore, increasing the motor complexity of resistance training exercises in older populations enhances functional capacity, which can transfer daily activities and independence (49).

**Figure 2:**
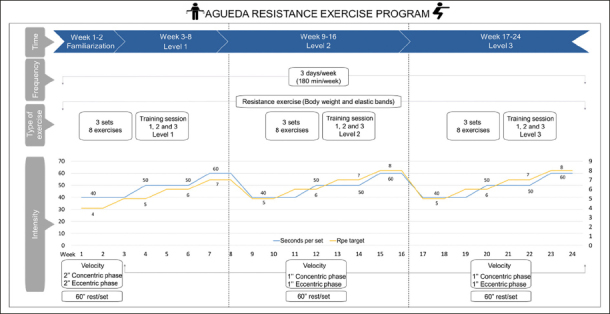
Characteristics and periodization of the supervised AGUEDA resistance exercise program

A summary of the type and load is presented in Figure 2. However, the total dose was quantified on an individualized and standardized basis as explained below.

#### Standardized load

Participants were prescribed 3 sets of 8 exercises per session. The duration of each set was from 40 to 60 seconds according to each week's assigned level with a rest of 60 seconds between sets. The resting time between exercises was adjusted to the instructions and preparation to the next exercise. The execution time equally increased at each level to ensure that each participant achieved the targeted intensity with a proper technique: 40 seconds per set execution within the first 3 weeks, 50 seconds of execution within the next 3 weeks and 60 seconds of execution within the 7th and 8th week. This equal number of sets and time allowed each participant to perform a certain number of repetitions according to their own capabilities. Velocity of the different exercises was used as another measure to modulate the intensity and adaptations at a muscular level, using as fast as possible but controlled execution speed (i.e., 1 sec of eccentric and 1 sec of concentric phase) (50). In addition, the maximum prescribed-target intensity was 70–80% of the participants' maximum rating of perceived exertion (7–8 RPE). RPE was recorded after each exercise and at the end of the entire session (47) (File A1). Participants started the exercise program within 2 weeks of familiarization. The familiarization period focused on exercise techniques described above using controlled execution speed and not on intensity (i.e., 2 sec of eccentric and 2 sec of concentric phase).

#### Individualized external load

It was important to consider the individual limitations and physical differences among participants to achieve the desired intensity, particularly in older populations. All participants started the exercise program with a soft elastic resistance (e.g., yellow-soft) for familiarization with the different exercises, but the resistance on the elastic bands increased on an individual basis. Elastic resistance was progressively increased at the judgement of the trainer and participant, aiming to reach the weekly target RPE, based on the standardized basis mentioned above.

### Structure of sessions and exercises

**Figure 3:**
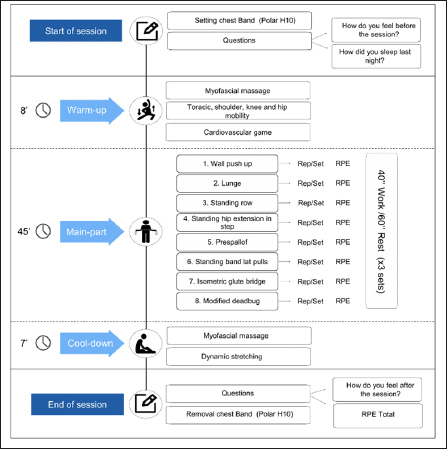
General structure of AGUEDA training session. Example of training session 1 for level 1 (week 1)

The exercise sessions included (i) a warm-up, (ii) the main part of resistance exercises and (iii) a cool-down phase (Figure 3). Data collected during the sessions were recorded in a paper-training group sheet during the session (i.e., date, duration of phases, and individualized data mentioned above) (File A2) and then, data were registered in an individual and group registration Excel sheet for all participants (File A3 and File A4). A detailed description of the AGUEDA exercise training program is shown in a multi-language manual and series of videos (File A5 and File A6).

Rep: Repetition; RPE: Rate of perceived exertion.

#### Warm-up

The warm-up lasted ∼8 min and included: (i) myofascial massage with foam roller or tennis ball, (ii) joint mobility (i.e., thoracic, shoulder, knee and hip mobility), and (iii) cardiovascular game (e.g., relay race).

#### Exercise

The duration of the main exercise intervention lasted about 45 min. There were 3 different exercise sessions per level, and each exercise session included 8 different exercises. The exercise progression from one exercise to another was done sequentially (i.e., 3 sets of exercise 1 – rest – 3 sets of exercise 2). Table 1 describes the exercises included in each exercise session and each level. The selected exercises included basic movement patterns (51) involving large muscle groups, horizontal traction, vertical traction, horizontal thrust, vertical thrust, hip extension and flexion, hip dominants, knee dominants, anti-rotation, anti-extension, anti-flexion, and antilateral flexion (52). Each session start with exercises in standing position (i.e., except training session 1 of level 3 that began with push-ups) and ended with specific lumbopelvic exercises in prone or supine position to avoid abrupt changes in body position that may cause dizzinesss (53).

#### Cool-down

The cool-down lasted approximately 7 min. This phase aimed for relaxation including myofascial release (54), joint mobility or stretching (i.e., static, or dynamic), and was focused on the trained muscle groups.

#### Modifications and adaptations of exercises

Exercise adaptation is an important factor when designing exercise programs, particularly in older adults (53, 55, 56). The AGUEDA exercise program included specific adaptations for the exercises due to different causes:

(i) Previous pain: the prevalence of pain in older adults increases with age (55). We added different modifications and adaptations for shoulder, knee, and back pain, such as unilateral exercises for the other painful limb (e.g., right, or left leg), isometric exercises for the painful muscles (e.g., isometric wall squat), and a change within the plane of movement (e.g., plank on wall).
(ii) Injury during the intervention: falls are the leading cause of injury among adults aged ≥65 years (56). After rehabilitation and/or recovery, participants were asked to rejoin the training sessions. The trainers modified and adapted the exercises under the supervision and clearance of a physician.
(iii) Vestibular symptoms and dizziness: these symptoms are a usual and significant problem in the elderly (53). Exercises that resulted in dizziness were modified by changing the exercise position from lying to standing (e.g., standing dead bug) or the exercise movement from dynamic to static (i.g., isometric squat instead of squat).

### Other variables recorded

The AGUEDA research team collected additional variables related to the training during all sessions:

(i) Participant heart rate (HR): HR was recorded using HR monitors (Chest strap Polar H10, Polar, Kempele, Finland) during all sessions for safety purposes to identify abnormal or excessive cardiovascular responses during exercise. The data was stored in an online training diary using 2 apps (Polar Flow and Elite HRV) for analyzing exercise intensity matching with RPE scale and further to understand the cardiovascular adaptations of this resistance exercise program. The validity of the Polar chest strap and the apps have been examined and described in previous studies (57–59). Each participant had a specific email account for apps to be associated to a specific mobile phone (Huawei MI A2 lite), which was carried by the participant throughout the session.
(ii) Participant feeling and sleep quality: feeling scale before and after each exercise session was measured by a validated question: “How do you feel before/after the session?” (60). In addition, previous night sleep quality was also reported by participants before each training session using an individual validated question: “How well did you sleep last night?” (61).

### Feasibility

Feasibility of AGUEDA project was evaluated by retention (62), adherence, adverse events (63, 64) (Table 2) and cost estimation on the exercise program (Table 3).

**Table 2:** Retention, adherence, and adverse event achieve for the AGUEDA trial

	**Previous criteria**	**Intervention group (N=46)**	Reason*
Retention	>90%	95.65%	N= 2 Health condition
Adherence	>80% attendance rate	≤59% N=2 60–80% N=3 >80% N=41	N=5 Health condition
	AMAP**	10% N=53	-
Adverse event	≤10% of serious adverse event	1%	Shoulder surgery


*All the reasons were external to the exercise program. **As much as possible. - No applicable

**Table 3:** Economic cost of the AGUEDA trial

	**Resource use**	**Unit cost**	**Individual cost**	**Group cost**	**Intervention cost (N=46)**
Human resources	1 trainer	8,12 €/ hour*	1,35 €/ hour**	8,12 €/ hour**	4471,24 €/ 24weeks
Equipment	8 elastics bands	17,81 €	17,81 €	106,86 €	819,26 €
	Mat	3,99 €	3,99 €	23,94 €	183,54 €
	Step	24,99 €	24,99 €	149.94 €	1149.54 €
Total cost	24 week	-	144.23 €	865.49 €	6623.58 €


*(Trainer salary*training hours per month/total working hours)/1 hour. **Based on an average group size of 6 participants

#### Retention

The retention was calculated from the number of participants from the intervention group who had completed the post assessments (65). Study retention was 95.65% (44 of 46 participants completed the post assessments).

#### Adherence

Adherence to the exercise program was measured by session attendance by the proportion of sessions completed out of the total prescribed sessions. An 80% attendance was required for the per protocol analysis (i.e., > 57 exercise sessions). Exercise sessions were performed on a regular basis during holidays (i.e., summer or Christmas). Any missed session was registered and rescheduled to an alternative date to maximize program adherence. For exceptional cases when rescheduling was not feasible, online sessions were conducted through a video call using the following materials: (i) mobile phone, (ii) Polar band H10, (iii) elastics bands, (iv) RPE scale, and (v) an online training program guide previously explained in detail (File A8).

The average recorded attendance of 46 participants was 84.12%. 10% (53 out of 454 sessions) of the missed sessions were rescheduled, increasing the average attendance to 85.71%. 2 participants had less than 60% of attendance, 3 participants between 60–80%, and 41 participants achieve >80%. External factors to the exercise program were identified as the cause for missed sessions. Reasons for the lowest adherence (<60% of attendance) were unwillingness to undertake the exercise program for the presence of a health condition. Regarding the others, the 3 participants missed sessions due to health conditions.

In addition, specific strategies were implemented to promote participant engagement:

(i) Music: Speaker with music during the exercise sessions based on the participant's choice.
(ii) Extrinsic motivation: well-internalized extrinsic motivation by the trainers in charge, such as personally valuing certain outcomes of the exercises as a particularly important factor for initial adoption (66) (e.g., “With this exercise you will gain muscle mass in your back”).
(iii) Intrinsic motivation: individual intrinsic feedback in a close and encouraging attitude by the trainers (67) (e.g., “Inhale and exhale slowly”).
(iv) Group feedback: positive group feedback in order to promote feelings of competence and self-confidence in the participants (e.g., “All of you have improved a lot, keep going”).
(v) Workshops: bimonthly body-mind, mobility or game workshops were held to keep motivation and maintain contact during the 24 wait-weeks with the wait-list control group.

Upon program completion, three additional strategies were used to help participants to continue engaging in exercise:

(i) Guide of the AGUEDA exercise program: a complete manual of the AGUEDA exercise program, including a multi-language visual and theoretical description of each of the training sessions, was delivered to participants to facilitate the practice of physical exercise in an autonomous way (File A5 and File A6).
(ii) Wait-list control group: Participants assigned to the waitlist control group could perform the exercise program after finishing the post-evaluations.
(iii) Flyer: An invitation, with a welcome discount, to become a member of a training center in Granada (Spain) to help participants (i.e., exercise and wait-list control group) to maintain their training habit under the supervision of a professional personal trainer (File A9) after finishing the project.

#### Adverse Event

Any adverse event that occurred, such as injury, emergency, or scheduled surgery, was recorded, reported, and evaluated by the research team in REDCap (68), an online platform designed to store and manage electronic data. Adverse data were recorded, if possible, at the time of the event but also asked at midpoint and post assessments (i.e., at 12 and 25 week) by a phone call. A customized adverse event form (File A7) included the seriousness, severity, chronicity, and resolution occurrence in participants, even events unrelated to the exercise program (e.g., COVID). The severity of adverse events was classified in 3 categories (i.e., mild, moderate, severe) (69). In case of joint injury or other injuries, a physician clearance was required before rejoining the resistance exercise program. There was 1 severe adverse event during the intervention period but without causal relationship with assessments or the exercise program.

#### Economic cost estimation

We report an estimation of the cost of delivering the AGUEDA exercise program including the human resources and equipment (Table S5). The cost is based on an average group size of 6 participants. The estimated total cost of the 24-week AGUEDA resistance training program was 6623.58 €, with an approximate cost of 144.23 € per participant and 865.49 € per group.

The costs have been calculated in based to the minimal material required for replication in any context. Benefits of body weight resistance training has been recognized by the ACSM by a functional way to exercise with minimal equipment and space, making it an inexpensive, convenient, and accessible form of exercise for people of all ages and fitness levels (70).

Equally the use of elastic bands is increasing as an alternative method for improve muscle strength (71, 72) in older adults because of the following reasons: (i) low-cost and available instead of weight machines (73–75), (ii) convenient to different levels of physical fitness (76) and (iii) portability; allowing individuals home or outdoor use (75).

## Discussion

Although evidence supports the potential role of resistance exercise on brain health during aging (1), there is insufficient information to allow replication of exercise programs and for determining the appropriate characteristics of resistance exercise for cognitive and brain health benefits in cognitively normal older adults (14). The present study has described a CERT-based description of the 24-week supervised resistance exercise program implemented in the AGUEDA trial, a RCT investigating the effects on brain health in cognitively normal older adults.

Previous literature has primarily focused on the effects of aerobic exercise (i.e., walking) on cognition (23, 77–80). However, the potential benefits of resistance training on cognitive functioning are being increasingly investigated for different reasons; (i) the greatest benefit of aerobic exercise on cognition seems to occur when it was combined with resistance training (81), (ii) evidence showed that resistance exercise has benefits on global cognitive function (4, 5, 19, 82), memory (19), executive function (4, 5, 15, 19), processing speed (18) and working memory (4), and (iii) there are physiological mechanisms by which resistance training might ameliorate cognitive function independently of aerobic exercise (83). In addition, a large body of evidence has shown positive effects of resistance exercise on others health indicators for older adults, such as functional capacity (28, 84, 85), increased muscular strength (85), and other behavioral outcomes such as depressive symptoms (86, 87), anxiety symptoms (86), and sleep (19) among others. Despite previous literature, there remains significant heterogeneity and insufficient information in terms of exercise characteristics that preclude its replication (1, 4, 5, 9, 19, 27), which is limiting the evidence about the characteristics-response effects of resistance exercise on brain health on specific cognitive domains (9). Moreover, there are few studies that have been performed in cognitively normal older adults, in comparison with populations with cognitive impaired, and high-quality reporting of exercise interventions focused on brain health is scarce.

The AGUEDA exercise program has been designed using ACSM guidelines for older adults with the emphasis on resistance exercise (46), as well as scientific literature demonstrating overall health benefits of resistance exercise for this population (9, 51, 88). The exercise program was performed with elastic bands and body weight, which is an effective strategy for improving basic movement patterns, instrumental activities of daily living (34), and for functional tasks related to safety when is perform with high speed (89). In addition, the exercises were based on specific movements, as pushing, pulling, lifting, holding, trunk flexion, trunk rotation and stabilization, making exercises more functional, efficient, and beneficial for increasing absolute strength and power in older adults (51, 90)

Furthermore, the current exercise program seems to be feasible in comparation with other interventions (91, 92), by the high retention (95.6%) and attendance rate (85.7%), minor adverse event (1%) and low economic cost (144.23 €/participant/24 weks) in comparation with other types of training (i.e., weight training equipment (73–75)). The AGUEDA program's accessibility and portability allow it to be performed anytime and anywhere, making it a feasible exercise program for older population (71). Its convenience and adaptability to different fitness levels and training goals make it easier replication, such as fitness centers, homes, or research laboratories (76). Moreover, we have carefully considered several adherence exercise strategies, based on social-cognitive principles (93), to maximize participant engagement to the AGUEDA exercise program.

### Limitations and strengths

The present study has several limitations. Recruitment started during COVID (i.e., March 2021) and was slower than expected, starting at different times of the year. This made it difficult to implement the 24-week exercise program at the same time of the year for all training groups. Seasonal exercising may not have impact on physical fitness (94), but weather should be considered when interpreting differences in physical activity patterns (95) including the use of a mask during the training sessions (96). However, the schedule was flexible and was largely directed by the participant's needs, and uninterrupted during the 24 weeks. Another limitation could be the previous experience and the physical condition of the participants, both being heterogeneous and potentially acting as moderators of any outcomes reported in the study. For example, prior experience with exercise could influence the individualized prescriptions and progress in the resistance and repetitions between participants.

There are also several strengths that must be acknowledged. First, a detailed description of the resistance exercise program was implemented which requires minimal equipment and enables application and translation to a different daily life context (e.g., elderly person at home) and clinical practices (e.g., physiotherapist). Second, the design included a resistance exercise program based on evidenced-based exercise guidelines for older adults (97). Third, the specific population focused on cognitively normal older adults who do not have a clear characteristics-response of exercise. Last, the program included a variety of exercises as well as adherence strategies that could increase adherence to the exercise programs.

## Conclusion

In conclusion, the comprehensive CERT-based description of the AGUEDA trial, including well-described multi-language manuals and videos, will serve researchers and public health practitioners who wish to implement a feasible and low-cost 24-week resistance exercise program in cognitively normal older adults.

## Electronic supplementary material

Supplementary material, approximately 702 KB.
