# Supplementary material for: Resistance Exercise Program in Cognitively Normal Older Adults: CERT-Based Exercise Protocol of the AGUEDA Randomized Controlled Trial
Source: J Nutr Health Aging. 2023 Sep 30;27(10):885–93. doi: 10.1007/s12603-023-1982-1 (PMC12880457; doi:10.1007/s12603-023-1982-1)
Supplement: Supplementary file 1 — Supplementary material, approximately 702 KB. [file mmc1.docx]

**Table S1:** CERT 16-item checklist

|  | Item | Checklist item | Identification |
| --- | --- | --- | --- |
| What: materials | 1 | Detailed description of the type of exercise equipment (e.g., weights, exercise equipment such as machines, treadmill, bicycle ergometer etc) | Exercise equipment |
| Who:provider | 2 | Detailed description of the qualifications, teaching/supervising expertise, and/or training undertaken by the exercise instructor | Exercise program structure |
| How:delivery | 3 | Describe whether exercises are performed individually or in a group | Exercise program structure |
|  | 4 | Describe whether exercises are supervised or unsupervised and how they are delivered | Exercise program structure |
|  | 5 | Detailed description of how adherence to exercise is measured and reported | Other variables recorded |
|  | 6 | Detailed description of motivation strategies | Feasibility |
|  | 7a | Detailed description of the decision rule(s) for determining exercise progression | Exercise doses |
|  | 7b | Detailed description of how the exercise program was progressed | Exercise doses |
|  | 8 | Detailed description of each exercise to enable replication (e.g., photographs, illustrations, video etc) | Structure of sessions and exercises |
|  | 9 | Detailed description of any home program component (e.g., other exercises, stretching etc) | Structure of sessions and exercises |
|  | 10 | Describe whether there are any non-exercise components (e.g., education, cognitive behavioural therapy, massage etc) | Other variables recorded  Feasibility |
|  | 11 | Describe the type and number of adverse events that occurred during exercise | Other variables recorded |
| Where: location | 12 | Describe the setting in which the exercises are performed | Exercise program structure |
| When, how much: dosage | 13 | Detailed description of the exercise intervention including, but not limited to, number of exercise repetitions/sets/sessions, session duration, intervention/program duration etc | Exercise characteristics and periodization |
| Tailoring: what, how | 14a | Describe whether the exercises are generic (1 size fits all) or tailored whether tailored to the individual | Standardized load  Individualized external load |
|  | 14b | Detailed description of how exercises are tailored to the individual | Individualized external loadModifications and adaptations of exercises |
|  | 15 | Describe the decision rule for determining the starting level at which people commence an exercise program (such as beginner, intermediate, advanced etc) | Standardized load  Individualized external load |
| How well: planned, actual | 16a | Describe how adherence or fidelity to the exercise intervention is assessed/measured | Feasibility |
|  | 16b | Describe the extent to which the intervention was delivered as planned | Feasibility |

Table S2. Additional files available in the repository

| Additional files* | Description |
| --- | --- |
| File A1 | [Registration sheet used by the participant](https://drive.google.com/file/d/1l6kJsW230oG6fow9C7dCqVJBwnCDCPVn/view?usp=share_link). |
| File A2 | [Training sheets](https://drive.google.com/drive/u/2/folders/1EAPYhLGzhkVUyfcQb2FxM_YHpXbeRabs) |
| File A3 | [Individual Excel for registration of exercise data](https://docs.google.com/spreadsheets/u/2/d/1mli3uBttadDveAWZWSi3Drk1ORUF0w-M/edit?usp=drive_web&ouid=110439011713121316633&rtpof=true). |
| File A4 | [Generic Excel for registration of exercise data](https://docs.google.com/spreadsheets/d/1UTFzm6OAY7RS9lyBN5F2LKfLN0uxXKxd/edit#gid=979997974) |
| File A5 | [Resistance exercise program of the AGUEDA trial: Paper guide](https://docs.google.com/document/d/1c8jkDGP-c0x6KOYPjrDNttxCAYWea8n3/edit#heading=h.gjdgxs) |
| File A6 | [Resistance exercise program of the AGUEDA trial: Video guide](https://drive.google.com/drive/u/1/folders/1N-VCTgdM07iVMo1FHnY-JV4JFon-1Z52) |
| File A7 | [Adverse event questionnaire](https://docs.google.com/document/d/1nkyuj2exz7eYJtm35oDy3VnAcsX0c7dM/edit) |
| File A8 | [Online exercise program guide](https://docs.google.com/document/d/1HgQxN0Yge_Dnq9ZaUnM65LWC8Pt89cWp/edit) |
| File A9 | [Flyer personal training center](https://drive.google.com/file/d/1iC4o7TS1mDzIp_HUJvm-vfmNUV7vUZGc/view?usp=sharing) |

*Avaliable on github (<https://github.com/aguedaprojectugr/CERT_AGUEDA>

Table S3. Exercise equipment for the AGUEDA trial.

| Image | Equipment | Number of items | Characteristics |
| --- | --- | --- | --- |
| 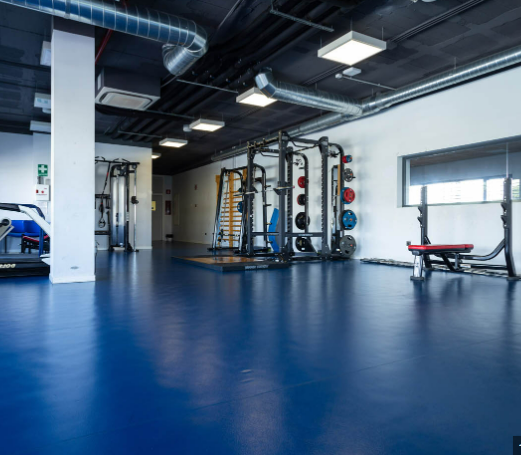 | Training room | 1 | 4m x 4m of larger  157,48 in x 147,48 in |
| 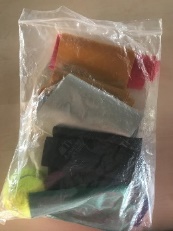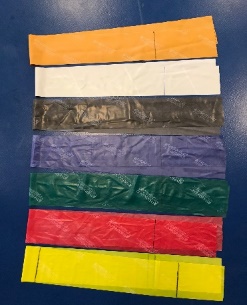 | Bag with elastics bands and tennis ball | 7+1 | 1. Yellow-Soft band 2. Red-Medium band 3. Green-Strong band 4. Blue -Extra Strong band 5. Black-Strong Special band 6. Silver-Athletic band 7. Gold-Olympic band   Classic tennis ball |
| 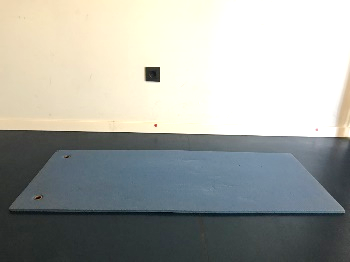 | Fitness mat | 1 | The dimensions of the mat are:   - 125 cm/49,21 in of long - 51 cm /20,07 in of wide - 6 mm/0,23 in of thickness |
| 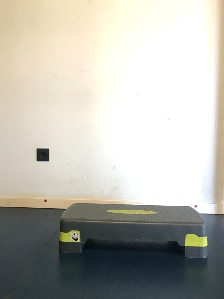 | Step | 1 | Aerobic Step Board Professional Elite with 10 cm/3,93 in of high |
| 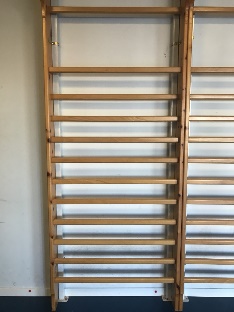 | Rack | 1 | 4 points of elastic support or similar support: at the knee, waist, head and above our head |
|  |  |  |  |

*Exercise equipment used per participant and one training session

Table S3. Exercise equipment for the AGUEDA trial (continued)

| 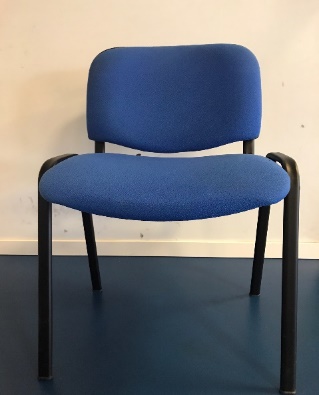 | Chair | 1 | The height of the chair is 45 cm |
| --- | --- | --- | --- |
| 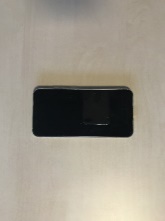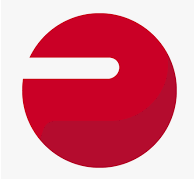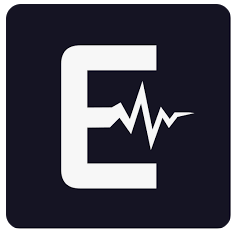 | Phone and applications | 1 + 2 | Huawei MI A2 lite phone with applications “Polar Beat” and “Elite HRV” |
| 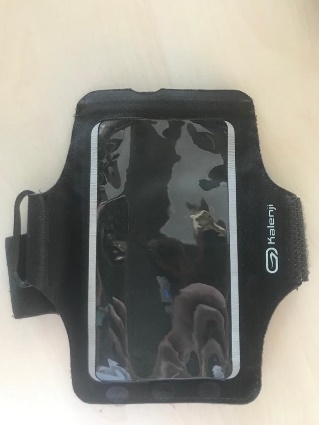 | Phone holder | 1 | Standard holder carried on the arm throughout the session |
| 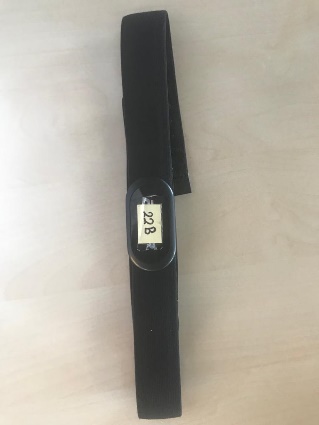 | Polar band h10 | 1 | Chest strap Polar H10, Polar, Kempele, Finland |
| 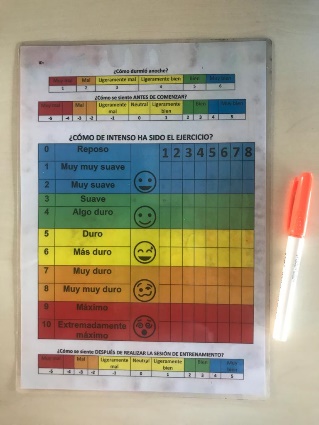 | RPE scale and marker | 1 | Laminated document for reuse and with sufficient font size |

Supplementary material 4. Additional references

41. Beaman SR de, Beaman PE, Garcia-Peña C, Villa MA, Heres J, Córdova A, et al. Validation of a Modified Version of the Mini-Mental State Examination (MMSE) in Spanish. Aging, Neuropsychology, and Cognition [Internet]. 2004 Mar 9;11(1):1–11. Available from: https://www.tandfonline.com/doi/full/10.1076/anec.11.1.1.29366

42. Ojeda N, del Pino R, Ibarretxe-Bilbao N, Schretlen DJ, Pena J. [Montreal Cognitive Assessment Test: normalization and standardization for Spanish population]. Rev Neurol [Internet]. 2016 Dec 1;63(11):488–96. Available from: http://www.ncbi.nlm.nih.gov/pubmed/27874165

43. Fernández-San Martín MI, Andrade C, Molina J, Muñoz PE, Carretero B, Rodríguez M, et al. Validation of the Spanish version of the geriatric depression scale (GDS) in primary care. Int J Geriatr Psychiatry. 2002;17(3):279–87.

44. Solis-Urra P, Molina-Hidalgo C, García-Rivero Y, Costa-Rodriguez C, Mora-Gonzalez J, Fernandez-Gamez B, et al. Active Gains in brain Using Exercise During Aging (AGUEDA): Protocol for a randomized controlled trial. Front Hum Neurosci [Internet]. 2023;17(1168549).
Available from: https://clinicaltrials.gov/ct2/show/NCT05186090

45. Izquierdo M, Merchant RA, Morley JE, Anker SD, Aprahamian I, Arai H, et al. International Exercise Recommendations in Older Adults (ICFSR): Expert Consensus Guidelines. Journal of Nutrition, Health and Aging. 2021 Jul 1;25(7):824–53.

46. Kraemer, W. J., Adams, K., Cafarelli, E., Dudley, G. A., Dooly, C., Feigenbaum, M. S., ... & Triplett-McBride T. Progression Models in Resistance Training for Healthy Adults. Med Sci Sports Exerc [Internet]. 2009 Mar;41(3):687–708. Available from: https://journals.lww.com/00005768-200903000-00026

47. Colado JC, Garcia-Masso X, Travis Triplett N, Calatayud J, Flandez J, Behm DG, et al. Construct and concurrent validation of a new resistance intensity scale for exercise with thera-band® elastic bands. J Sports Sci Med. 2014;13(4):758–66.

48. Morishita S, Tsubaki A, Nakamura M, Nashimoto S, Fu JB, Onishi H. Rating of perceived exertion on resistance training in elderly subjects. Vol. 17, Expert Review of Cardiovascular Therapy. Taylor and Francis Ltd; 2019. p. 135–42.

49. La Scala Teixeira C V., Evangelista AL, Pereira PE de A, Da Silva-Grigoletto ME, Bocalini DS, Behm DG. Complexity: A Novel Load Progression Strategy in Strength Training. Front Physiol. 2019 Jul 3;10.

50. Da Rosa Orssatto LB, Cadore EL, Andersen LL, Diefenthaeler F. Why fast velocity resistance training should be prioritized for elderly people. Strength Cond J. 2019;41(1):105–14.

51. Ribeiro AS, Nunes JP, Schoenfeld BJ. Selection of Resistance Exercises for Older Individuals: The Forgotten Variable. Sports Medicine [Internet]. 2020 Jun 1;50(6):1051–7. Available from: https://doi.org/10.1007/s40279-020-01260-5

52. Page P, Ellenbecker T. Strength Band Training. Vol. 60, Physiotherapy Canada. 2008. 195 p.

53. Balatsouras DG, Koukoutsis G, Fassolis A, Moukos A, Apris A. Benign paroxysmal positional vertigo in the elderly: Current insights. Clin Interv Aging. 2018;13:2251–66.

54. Cheatham SW, Kolber MJ, Cain M, Lee M. The effects of self-myofascial release using a foam roll or roller massager on joint range of motion, muscle recovery, and performance: A systematic review. Int J Sports Phys Ther [Internet]. 2015 Nov;10(6):827–38. Available from: http://www.ncbi.nlm.nih.gov/pubmed/26618062

55. Abdulla A, Adams N, Bone M, Elliott AM, Gaffin J, Jones D, et al. Guidance on the management of pain in older people. Age Ageing [Internet]. 2013 Mar 1;42(suppl 1):i1–57. Available from: https://academic.oup.com/ageing/article-lookup/doi/10.1093/ageing/afs200

56. Moreland B, Kakara R, Henry A. Trends in Nonfatal Falls and Fall-Related Injuries Among Adults Aged ≥65 Years — United States, 2012–2018. MMWR Morb Mortal Wkly Rep. 2020;69(27):875–81.

57. Moya-Ramon M, Mateo-March M, Peña-González I, Zabala M, Javaloyes A. Validity and reliability of different smartphones applications to measure HRV during short and ultra-short measurements in elite athletes. Comput Methods Programs Biomed [Internet]. 2022;217:106696. Available from: https://doi.org/10.1016/j.cmpb.2022.106696

58. Müller AM, Wang NX, Yao J, Tan CS, Low ICC, Lim N, et al. Heart rate measures from wrist-worn activity trackers in a laboratory and free-living setting: Validation study. JMIR Mhealth Uhealth. 2019;7(10):1–19.

59. Gilgen-Ammann R, Schweizer T, Wyss T. RR interval signal quality of a heart rate monitor and an ECG Holter at rest and during exercise. Eur J Appl Physiol [Internet]. 2019;119(7):1525–32. Available from: https://doi.org/10.1007/s00421-019-04142-5

60. Hardy CJ, Rejeski WJ. Not What, but How One Feels: The Measurement of Affect during Exercise. J Sport Exerc Psychol [Internet]. 1989 Sep;11(3):304–17. Available from: https://journals.humankinetics.com/view/journals/jsep/11/3/article-p304.xml

61. Ellis BW, Johns MW, Lancaster R, Raptopoulos P, Angelopoulos N, Priest RG. The St. Mary’s Hospital Sleep Questionnaire: A Study of Reliability. Sleep [Internet]. 1981 Sep;4(1):93–7. Available from: https://academic.oup.com/sleep/article-lookup/doi/10.1093/sleep/4.1.93

62. Sow LC, Liu HH, Wang RY, Wei SH, Wu HK, Yang YR. Feasibility and effectiveness of interactive stepping exercise on community-dwelling older adults: A pilot randomized controlled trial. Geriatr Nurs (Minneap). 2021 Sep 1;42(5):1099–104.

63. Ptomey LT, Vidoni ED, Montenegro-Montenegro E, Thompson MA, Sherman JR, Gorczyca AM, et al. The feasibility of remotely delivered exercise session in adults with Alzheimer’s disease and their caregivers. J Aging Phys Act. 2019;27(5):670–7.

64. Suttanon P, Hill KD, Said CM, Williams SB, Byrne KN, Logiudice D, et al. Feasibility, safety and preliminary evidence of the effectiveness of a home-based exercise programme for older people with Alzheimer’s disease: A pilot randomized controlled trial. Clin Rehabil. 2013 May 1;27(5):427–38.

65. Daly RM, Gianoudis J, Hall T, Mundell NL, Maddison R. Feasibility, usability, and enjoyment of a home-based exercise program delivered via an exercise app for musculoskeletal health in community-dwelling older adults: Short-term prospective pilot study. Vol. 9, JMIR mHealth and uHealth. JMIR Publications Inc.; 2021.

66. Teixeira PJ, Carraça E v, Markland D, Silva MN, Ryan RM. Exercise, physical activity, and self-determination theory: a systematic review. Int J Behav Nutr Phys Act [Internet]. 2012 Jun 22;9:78. Available from: http://www.ncbi.nlm.nih.gov/pubmed/22726453

67. Shin M, Kim I, Kwon S. Effect of intrinsic motivation on affective responses during and after exercise: latent curve model analysis. Percept Mot Skills [Internet]. 2014 Dec;119(3):717–30. Available from: http://www.ncbi.nlm.nih.gov/pubmed/25456247

68. Harris PA, Taylor R, Minor BL, Elliott V, Fernandez M, O’Neal L, et al. The REDCap consortium: Building an international community of software platform partners. J Biomed Inform [Internet]. 2019 Jul 1;95:103208. Available from: http://www.ncbi.nlm.nih.gov/pubmed/31078660

69. Freites-Martinez A, Santana N, Arias-Santiago S, Viera A. CTCAE versión 5.0. Evaluación de la gravedad de los eventos adversos dermatológicos de las terapias antineoplásicas. Actas Dermosifiliogr [Internet]. 2021 Jan 1;112(1):90–2. Available from: https://linkinghub.elsevier.com/retrieve/pii/S0001731020302866

70. Thompson WR. Worldwide Survey of Fitness Trendsfor 2023 [Internet]. 2023. Available from: www.acsm-healthfitness.org

71. Davis NM, Pringle A, Kay AD, Blazevich AJ, Teskey D, Faghy MA, et al. Feasibility, Psychosocial Effects, Influence, and Perception of Elastic Band Resistance Balance Training in Older Adults. Int J Environ Res Public Health. 2022 Sep 1;19(17).

72. De Oliveira PA, Blasczyk JC, Junior GS, Lagoa KF, Soares M, De Oliveira RJ, et al. Effects of Elastic Resistance Exercise on muscle strength and functional performance in healthy adults: A systematic review and meta-analysis. Vol. 14, Journal of Physical Activity and Health. Human Kinetics Publishers Inc.; 2017. p. 317–27.

73. Martins WR, Safons MP, Bottaro M, Blasczyk JC, Diniz LR, Fonseca RMC, et al. Effects of short term elastic resistance training on muscle mass and strength in untrained older adults: a randomized clinical trial. BMC Geriatr. 2015 Aug 12;15(1):1–10.

74. Colado JC, Garcia-Masso X, Pellicer M, Alakhdar Y, Benavent J, Cabeza-Ruiz R. A comparison of elastic tubing and isotonic resistance exercises. Int J Sports Med. 2010;31(11):810–7.

75. Colado JC, Triplett NT. EFFECTS OF A SHORT-TERM RESISTANCE PROGRAM USING ELASTIC BANDS VERSUS WEIGHT MACHINES FOR SEDENTARY MIDDLE-AGED WOMEN [Internet]. Available from: www.nsca-jscr.org

76. Chen KM, Tseng WS, Huang HT, Li CH. Development and feasibility of a senior elastic band exercise program for aged adults: A descriptive evaluation survey. J Manipulative Physiol Ther. 2013 Oct;36(8):505–12.

77. Mendez Colmenares A, Voss MW, Fanning J, Salerno EA, Gothe NP, Thomas ML, et al. White matter plasticity in healthy older adults: The effects of aerobic exercise. Neuroimage. 2021 Oct 1;239.

78. Erickson KI, Grove GA, Burns JM, Hillman CH, Kramer AF, McAuley E, et al. Investigating Gains in Neurocognition in an Intervention Trial of Exercise (IGNITE): Protocol. Contemp Clin Trials [Internet]. 2019 Oct;85:105832. Available from: https://linkinghub.elsevier.com/retrieve/pii/S1551714419305476

79. Stillman CM, Esteban-Cornejo I, Brown B, Bender CM, Erickson KI. Effects of Exercise on Brain and Cognition Across Age Groups and Health States. Trends Neurosci [Internet]. 2020;43(7):533–43. Available from: https://doi.org/10.1016/j.tins.2020.04.010

80. Morris JK, Vidoni ED, Johnson DK, van Sciver A, Mahnken JD, Honea RA, et al. Aerobic exercise for Alzheimer’s disease: A randomized controlled pilot trial. PLoS One. 2017 Feb 1;12(2).

81. Colcombe S, Kramer AF. Fitness Effects on the Cognitive Function of Older Adults. Psychol Sci [Internet]. 2003 Mar 25;14(2):125–30. Available from: http://journals.sagepub.com/doi/10.1111/1467-9280.t01-1-01430

82. Coelho-Júnior HJ, Gonçalves I de O, Sampaio RAC, Sampaio PYS, Cadore EL, Calvani R, et al. Effects of combined resistance and power training on cognitive function in older women: A randomized controlled trial. Int J Environ Res Public Health. 2020 May 2;17(10).

83. Liu-Ambrose T, Donaldson MG. Exercise and cognition in older adults: Is there a role for resistance training programmes? Vol. 43, British Journal of Sports Medicine. 2009. p. 25–7.

84. Casas-herrero Á, Asteasu MLS de, Antón-rodrigo I, Sánchez-sánchez JL, Montero-odasso M, Marín-epelde I, et al. Effects of Vivifrail multicomponent intervention on functional capacity : a multicentre , randomized controlled trial. J Cachexia Sarcopenia Muscle. 2022;13(February):884–93.

85. Schott N, Johnen B, Holfelder B. Effects of free weights and machine training on muscular strength in high-functioning older adults. Exp Gerontol [Internet]. 2019 Jul;122(December 2018):15–24. Available from: https://doi.org/10.1016/j.exger.2019.03.012

86. Cunha PM, Werneck AO, Nunes JP, Stubbs B, Schuch FB, Kunevaliki G, et al. Resistance training reduces depressive and anxiety symptoms in older women: a pilot study. Aging Ment Health [Internet]. 2021;26(6):1136–42. Available from: https://doi.org/10.1080/13607863.2021.1922603

87. Moraes HS, Silveira HS, Oliveira NA, Matta Mello Portugal E, Araújo NB, Vasques PE, et al. Is Strength Training as Effective as Aerobic Training for Depression in Older Adults? A Randomized Controlled Trial. Neuropsychobiology. 2020;79(2):141–9.

88. Cretnik K. The Effect of Eccentric vs . Traditional Resistance Exercise on Muscle Strength , Body Composition , and Functional Performance in Older Adults : A Systematic Review With. Frontiers in Sport and Active Living. 2022;4(April).

89. Pauff SM, Miller SC. High-speed power training in older adults: A shift of the external resistance at which peak power is produced. J Strength Cond Res [Internet]. 2014;78(2):711–6. Available from: https://www.ncbi.nlm.nih.gov/pmc/articles/PMC3624763/pdf/nihms412728.pdf

90. Mira PAC, Filho MM, Venturini GRO, Moreira OC, Leita L, Castro JBP de, et al. Effects of Different Types of Resistance Training and Detraining on Functional Capacity , Muscle Strength , and Power in Older Women : A Randomized Controlled Study. The journal of Strenght and conditioning Research. 2022;984–90.

91. van der Bij A. Effectiveness of physical activity interventions for older adults a review. Am J Prev Med [Internet]. 2002 Feb;22(2):120–33. Available from: https://linkinghub.elsevier.com/retrieve/pii/S0749379701004135

92. Marshall-McKenna R, Campbell E, Ho F, Banger M, Ireland J, Rowe P, et al. Resistance exercise training at different loads in frail and healthy older adults: A randomised feasibility trial. Exp Gerontol. 2021 Oct 1;153.

93. Cook DA, Artino AR. Motivation to learn: an overview of contemporary theories. Med Educ. 2016 Oct 1;50(10):997–1014.

94. Bezerra P, Rodrigues LP, Ayan C, Cancela JM. The influence of winter and summer seasons on physical fitness in aged population. Arch Gerontol Geriatr. 2018;76(February):80–4.

95. Park JH, Kim Y, Welk GJ, Silva P, Lee JM. Association with temperature variability and physical activity, sedentary behavior, and sleep in a free-living population. Int J Environ Res Public Health. 2021;18(24).

96. Epstein D, Korytny A, Isenberg Y, Marcusohn E, Zukermann R, Bishop B, et al. Return to training in the COVID-19 era: The physiological effects of face masks during exercise. Scand J Med Sci Sports. 2021 Jan 1;31(1):70–5.

97. Montero-Odasso M, van der Velde N, Martin FC, Petrovic M, Tan MP, Ryg J, et al. World guidelines for falls prevention and management for older adults: a global initiative. Age Ageing [Internet]. 2022 Sep 2;51(9). Available from: https://academic.oup.com/ageing/article/doi/10.1093/ageing/afac205/6730755
